# Limitations of representation learning in small molecule property prediction

**DOI:** 10.1038/s41467-023-41967-3

**Published:** 2023-10-13

**Authors:** Ana Laura Dias, Latimah Bustillo, Tiago Rodrigues

**Affiliations:** Research Institute for Medicines (iMed), Faculdade de Farmácia, Universidade de Lisboa, Lisbon, Portugal

**Keywords:** Cheminformatics, Computational chemistry

## Abstract

Machine learning is a powerful tool for the study and design of molecules. Here the authors comment a recent publication in *Nature Communications* which highlights the challenges of different molecular representations for data-driven property predictions.

## Teaser

Representation learning is making inroads into drug discovery. A study in *Nature Communications* emphasizes multiple limitations in property prediction. The results suggest that continued research and improvements are required for this specific area that coalesces machine learning and molecular medicine.

Biological and medicinal chemistry are experiencing an unprecedented (r)evolution with the emergence of machine learning (ML) algorithms, improved hardware, and data storage capabilities. The prime goal of ML in drug discovery is accelerating research by prioritizing the most relevant experiments, mitigating attrition from an early stage, and thus expediting development pipelines^1,2^. While of utmost relevance, the more involved cheminformatics community is now realizing that advanced deep learning algorithms rarely display desirable performance in multiple molecular design tasks involving the prediction of physicochemical and biological endpoints^3–5^. In fact, traditional ML algorithms and molecular representations are still the state-of-the-art, performance-wise, and may remain so as long as training data is scarce^4^. This is the case because deep learning algorithms are typically data hungry, i.e., requiring large amounts of high-quality data to train thousands to millions of parameters / weights that lead to an optimal model fit.

Drug discovery is a peculiar and challenging use case for discriminative ML models for several reasons: (1) high-throughput experimentation is available^6^ yet data scarcity is still the norm for real-world problems^7,8^; (2) sparse coverage of search spaces^5^, which impose data distribution shifts over the project timeline and concerns over the models’ domains of applicability; (3) experimental uncertainty is largely unaccounted for in ML models and a clear solution to this limitation is currently unavailable^9^. The latter is particularly concerning since it directly impacts the quality of the available training datasets, benchmarks, and the attainability of robust decision-making processes. Moreover, it is also apparent a persisting lack of standardized reporting practices in ML studies that make method comparison nontrivial and potentially misleading^9,10^. While we^9^ and others^11,12^ have suggested solutions and guidelines to overcome those issues, said guidelines are rooted on hands-on experience and are still not widely adopted. Building on that, Wang and co-workers^13^ go one step further and exemplify good ML practice with the widely used MoleculeNet data. MoleculeNet^14^ is not free of its own limitations as the dynamic range in some endpoints is irrelevant in a drug discovery setting. This suggests that better benchmarks are required. Still, the team exposed shortcomings of deep learning algorithms that should dampen unfounded hype around ML with molecular featurization based on graphs or natural language.

In a thorough methodology survey, the research team studied different factors that might bias method comparison and performance, such as input data, train/test splits, molecular representations, performance metrics and the random seed. More specifically, random forests (RF), extreme gradient boosting (XGBoost) and support vector machines (SVMs) were employed with circular fingerprints, to obtain relevant baseline models. Those models were pitted against a recurrent neural network, different flavors of transformers (e.g., MolBERT, GROVER), generative and graph-based methods that sieved directly through SMILES strings to learn a chemical language or graph descriptors. Despite pre-training routines, it became apparent that baseline models performed competitively or seemingly better in select bioactivity and physicochemical property datasets. In particular, RFs displayed the best performance on the BACE, BBBP, ESOL and Lipop use cases, which can be ascribed not only to the fingerprint descriptors, but also to the performance superiority of this type of algorithm in the low data regimes. Conversely, deep learning algorithms only became competitive in the HIV dataset, and in the prediction of molecular weight and number of atoms when datasets contained >1000 training examples. Albeit previously reported, the result further reinforces deficiencies in representation learning as a generally applicable solution to accelerate molecular medicine. An identical low performance pattern was observed when using scaffold splits to assess the model generalization on both unseen scaffolds and activity cliff molecules. In this case, the result was not entirely unexpected. One can speculate the reason lies not only on the customary low abundance of training data, but also on a data shift issue. In fact, the application of learning algorithms to previously unseen scaffolds likely imposes a distribution mismatch and a higher likelihood of mispredictions. This mismatch is often encountered in real-world drug discovery programs as molecular design can change dramatically over a project timeline. Experimentally, testing of chemical entities that significantly differ from prior knowledge can increase attrition, akin to using models outside their domain of applicability. It is thus understandable that learning algorithms underperform with scaffold splits, in comparison to random splits, where no development timelines are taken into account in the splitting routine.

When analyzing RFs, it was also found that no descriptor set works satisfactorily well on all predictive tasks, indicating that feature engineering and the development of molecular representation toolkits are and will continue being a current topic in computational medicinal chemistry. Another particularly interesting issue discussed by Wang and colleagues is the empirical binning of continuous bioactivity readouts – with enormous loss of information – to obtain classifiers rather than regressors. Arguably, the latter need more training data, which is sometimes incompatible with the experimentation costs. In the case of classifiers, it is also discussed the uneven (or so-called imbalanced) label distribution and the most appropriate metrics for model assessment to avoid erroneous or skewed comparisons^15^. As noted, the area under the receiver operating characteristic curve is commonly used to gauge performance in classifiers. However, it can be optimistic in imbalanced label distributions. In such scenarios, the precision–recall curve is advisable as it focuses on the minority class.

Overall, the team highlights numerous methodological shortcomings in ML toolkits and practices that the community as a whole must strive to change. Further, they speculate that self-supervised learning can bypass the need for human annotations and expensive experimentation, and hint that the contrastive type of self-supervised learning might be applicable to small datasets in drug discovery. Indeed, the presented data partly counter cycles the current enthusiasm in deep learning by showing that tree-based methods with fixed representations are likely still the best option for property prediction. Albeit surprising to some, the report by Wang and team should further spur investigations in a quest to make representation learning more competitive and suited to real-world molecular medicine.

## References

[1] Vamathevan J (2019). Applications of machine learning in drug discovery and development. Nat. Rev. Drug Discov..

[2] de Almeida AF, Moreira R, Rodrigues T (2019). Synthetic organic chemistry driven by artificial intelligence. Nat. Rev. Chem..

[3] Van Tilborg D, Alenicheva A, Grisoni F (2022). Exposing the limitations of molecular machine learning with activity cliffs. J. Chem. Inf. Model..

[4] Janela T, Bajorath J (2022). Simple nearest-neighbour analysis meets the accuracy of compound potency predictions using complex machine learning models. Nat. Mach. Intell..

[5] Saebi M (2023). On the use of real-world datasets for reaction yield prediction. Chem. Sci..

[6] Ahneman DT, Estrada JG, Lin S, Dreher SD, Doyle AG (2018). Predicting reaction performance in C–N cross-coupling using machine learning. Science.

[7] Shields BJ (2021). Bayesian reaction optimization as a tool for chemical synthesis. Nature.

[8] Reker D, Hoyt EA, Bernardes GJL, Rodrigues T (2020). Adaptive optimization of chemical reactions with minimal experimental information. Cell Rep. Phys. Sci..

[9] Bender A (2022). Evaluation guidelines for machine learning tools in the chemical sciences. Nat. Rev. Chem..

[10] Rodrigues T (2019). The good, the bad, and the ugly in chemical and biological data for machine learning. Drug Discov. Today Technol..

[11] Artrith N (2021). Best practices in machine learning for chemistry. Nat. Chem..

[12] Keeping checks on machine learning. *Nat. Methods* **18**, 1119–1119 (2021). 10.1038/s41592-021-01300-6 34608320

[13] Deng, J. et al. A systematic study of key elements underlying molecular property prediction. *Nat. Commun*. 10.1038/s41467-023-41948-6 (2023). 10.1038/s41467-023-41948-6 PMC10575948 37833262

[14] Wu Z (2018). MoleculeNet: a benchmark for molecular machine learning. Chem. Sci..

[15] Lee K (2021). Combating small-molecule aggregation with machine learning. Cell Rep. Phys. Sci..

